# Dr. Charles H. Sanford of New York Tells Why He Likes the Gwathmey Gas-Oxygen Apparatus

**Published:** 1917-04

**Authors:** Chas. H. Sanford

**Affiliations:** 109 East Eightieth St., New York


### Abstracts and Selections.

### Dr. Charles H. Sanford of New York Tells Why He Likes the Gwathmey Gas-Oxygen Apparatus.

NEW YORK, February 12, 1917.

*Dr. R. v. Foregger, The Foregger Co., 33 West Forty-second St., New York, N. Y.*

MY DEAR DR. FOREGGER: I am in receipt of your letter in which you ask me for my opinion regarding the often-passed remark, that it requires an expert to administer gas-oxygen and that this is the reason why many are reluctant to adopt an apparatus, even one as simple and practical as the Gwathmey.

Now, I know from experience in teaching anesthesia to hospital internes, that it is far more difficult for these students of anesthesia to grasp the principles of gas-oxygen administration than it is to teach them the use of ether-chloroform or anesthol or any combination of these.

This is due in my opinion partly to the apparatus used but principally to the subtlety of the gas and oxygen—the quickly changing degree of the depth of anesthesia, which confuses them. The patient becomes cyanotic more quickly and on goes the oxygen with a rush, result: patient out and kicking—then comes a rush of gas and the patient goes under and gets blue.

This is the case with all beginners and until I can get them to administer a steady even flow of the gases they have the trouble. They had this trouble with the older methods and appliances of administration when they had no guide for approximating the quantities of each gas used. I, therefore, looked about for a simple apparatus with some sort of an indicator which would give them a reliable basis for observing the flow of gas and oxygen which can only be adjusted by empirical judgment. For my part, I do not care whether this approximation is in terms of liters or in terms of holes in a water sight feed, but in view of breaking in new men on the subject I must give preference to the indicator which is the most reliable and least deceiving. After all the patient is the barometer, and his needs must be satisfied regardless of any printed schedule of amounts to be used, although these too have their use and scientific or economic value, particularly when we come to measure the least amounts used with the best results in any one case.

It can readily be seen that an anesthesia apparatus which can be set to deliver a steady volume of each of the two component ingredients must have the guiding hand of an anesthetist who has learned how to use these ingredients. No apparatus will of itself regulate the quantities automatically to meet the varying conditions of the patient, nor can the proportions or amounts required by the individual be figured and set in advance.

The simplest apparatus, therefore, it seemed to me, would be one shorn of all mechanical gauges, percentage scales, mixing chambers or any complications that tend to confuse the beginner and the average general anesthetist. Such an apparatus would be in service most of the time and would be simple enough for anyone to use; in other words, produce the simplest apparatus in which control of all the ingredients in a gas-oxygen and ether sequence may be had by one valve and one sight feed for each.

That is the reason I chose the Gwathmey apparatus for our hospital and for my private work, and I have not been disappointed.

And I do believe that your apparatus will stand the trial of all those who had their disappointment with other apparatus.

Sincerely yours,

CHAS. H. SANFORD, M. D.,
109 East Eightieth St., New York.

